# Committees Appointed at the Meeting of the American Medical Association in May, 1849

**Published:** 1849-12

**Authors:** 


*Committees appointed at the Meeting of the American Medical Association in May,* 1849.—We have given, in a previous number, the names of the Chairmen of the several Committees appointed at the last meeting of the American Medical Association. The following is a list of all the members composing said committeer, respectively:

*Committee on Medical Science.*

Dr. Usher Parsons, Providence, R. I., Chairman.

Dr. J. Bigelow, Boston.
" J. B. S. Jackson, Boston.
" A. B. Malcolm, Dubuque, Iowa.
Dr. Jas. Moultrie, Charleston, S. C.
" G. Emerson, Philadelphia.
" D. King, Newport, R. I.

*Committee on Practical Medicine.*

Dr. J. K. Mitchell, Philadelphia, Chairman.

Dr. R. La Roche, Philadelphia.
" F. West, Philadelphia.
" J. A. Sweet, New York.
Dr. James Jones, New Orleans.
" R. D. Arnold, Savannah.
" —— Smith, Indiana.

*Committee on Surgery.*

Dr. R. D. Mussey, Cincinnati, Chairman.

Dr. W. M. Awl, Columbus, Ohio.
" A. B. Shipman, Syracuse, N. Y.
" G. Fox, Philadelphia.
Dr. L. A. Dugas, Augusta, Ga.
" S. Parkman, Boston.
" J. R. Wood, New York.

*Committee on Obstetrics.*

Dr. T. G. Prioleau, Charleston, S. C., Chairman.

Dr. L. D. Ford, Augusta, Ga.
" Robert Lebby, Charleston, S. C.
" Josiah Bartlett, Stratham, N. H.
Dr. H. F. Askew, Wilmington, Del.
" John Evans, Chicago, Ill.
" Isaac Lincoln, Brunswick, Me.

*Committee on Medical Education.*

Dr. J. Roby, Baltimore, M. D. Chairman.

Dr. Blatchford, Troy, N. Y.
" G. C. M. Roberts, Baltimore.
" R. W. Silvester, Norfolk, Va.
Dr. F. A. Ramsey, Knoxville, Tenn.
" Geo. Sumner, Hartford, Conn.
" W. H. Rockwell, Brattleboro, Vt.

*Committee on Medical Literature.*

Dr. Alfred Stillé, Philadelphia, Chairman.

Dr. F. G. Smith, Philadelphia.
" T. H. Yardley, Philadelphia.
" P. C. Gaillard, Charleston, S. C.
Dr. A. T. Morris, Montgomery, Ala.
" J. Fithian, Woodbury, N. J.
" J. B. Johnson, St. Louis, Mo.

*Committee on Publication.*

Dr. I. Hays, Philadelphia, Chairman.

Dr. A. Stille, Philadelphia.
" H. J. Bowditch, Boston.
" D. F. Condie, Philadelphia.
Dr. B. F. Barker, Norwich, Conn.
" Isaac Wood, New Yord.
" N. J. Pittman, Rocky Mt., N. C.

*Committee on Forensic Medicine.*

Dr. A. H. Stevens, N. Y., Chairman.

| | |
|---|---|
| Dr. Luther V. Bell, Boston. | Dr. Robert Watts, New York. |
| " Pliny Earle, New York. | " R. S. Steuart, Baltimore. |
| " W. H. Rockwell, Vt. | " ,J. Knight, New Haven, Conn. |

*Coommittee on Indigenous Botany and Materia Medica.*

Dr. Eli Ives, New Haven, Chairman.

| | |
|---|---|
| Dr. G. L. Corbyn, Warwick Co. Va. | Dr. B. B. Lenoir, Roanne Co. Tenn. |
| " H. R. Frost, Charleston, S. C. | " W. B. Cochran, Middleburg, Va. |
| " W. H. Davis, Baltimore. | " J. P. Harrison, Cincinnati. |

*Committee on Hygiene.*

Dr. J. M. Smith, N. Y., Chairman.

| | |
|---|---|
| Dr. A. K. Gardner, New York. | Dr. R. S. Holmes, St. Louis, Mo. |
| " E. Jarvis, Dorchester, Mass. | " G. Emerson, Philadelphia. |
| " A. T. M. Cooke, Norfolk, Va. | " J. C. Simonds, New Orleans. |

